# Epigenetics of Modified DNA Bases: 5-Methylcytosine and Beyond

**DOI:** 10.3389/fgene.2018.00640

**Published:** 2018-12-18

**Authors:** Suresh Kumar, Viswanathan Chinnusamy, Trilochan Mohapatra

**Affiliations:** ^1^Division of Biochemistry, Indian Agricultural Research Institute (ICAR), New Delhi, India; ^2^Division of Plant Physiology, Indian Agricultural Research Institute (ICAR), New Delhi, India; ^3^Indian Council of Agricultural Research, New Delhi, India

**Keywords:** cytosine methylation, DNA modification, epigenetic marks, modified DNA base, 5-hydroxymethylcytosine, 5-methylcytosine, N^6^-methyladenine

## Abstract

Modification of DNA bases plays vital roles in the epigenetic control of gene expression in both animals and plants. Though much attention is given to the conventional epigenetic signature 5-methylcytosine (5-mC), the field of epigenetics is attracting increased scientific interest through the discovery of additional modifications of DNA bases and their roles in controlling gene expression. Theoretically, each of the DNA bases can be modified; however, modifications of cytosine and adenine only are known so far. This review focuses on the recent findings of the well-studied cytosine modifications and yet poorly characterized adenine modification which serve as an additional layer of epigenetic regulation in animals and discuss their potential roles in plants. Cytosine modification at symmetric (CG, CHG) and asymmetric (CHH) contexts is a key epigenetic feature. In addition to the ROS1 family mediated demethylation, Ten-Eleven Translocation family proteins-mediated hydroxylation of 5-mC to 5-hydroxymethylcytosine as additional active demethylation pathway are also discussed. The epigenetic marks are known to be associated with the regulation of several cellular and developmental processes, pluripotency of stem cells, neuron cell development, and tumor development in animals. Therefore, the most recently discovered N^6^-methyladenine, an additional epigenetic mark with regulatory potential, is also described. Interestingly, these newly discovered modifications are also found in the genomes which lack canonical 5-mC, signifying their independent epigenetic functions. These modified DNA bases are considered to be important players in epigenomics. The potential for combinatorial interaction among the known modified DNA bases suggests that epigenetic codon is likely to be substantially more complicated than it is thought today.

## Introduction

Modification of DNA base is one of the main epigenetic mechanisms which regulate gene expression in both plants and animals. DNA methylation indicates attachment of a methyl (CH_3_) group at 5′-carbon of pyrimidine ring of cytosine nucleotide. In addition to methylation of cytosine residue, other modifications such as oxidation of methylated cytosine (5-mC) to 5-hydroxymethylcytosine (5-hmC), 5-formylcytosine (5-fC), 5-carboxylcytosine (5-caC), and methylation of adenine (A) to N^6^-methyladenine (6-mA), are being identified as important epigenetic regulators (Klungland and Robertson, 2017). Supplementary epigenetic diversity in regulating gene expression is accomplished by post-translational modifications of histone proteins of the nucleosome. These modifications regulate cellular machinery to modulate specific chromatin regions and mark the regions for various cellular functions. The sum total of the chemical modifications in the nuclear DNA and N-terminal tail of histone proteins constitute epigenome. Epigenetics is a branch of functional genomics which deals with regulation of gene expression through DNA base and histone modifications (Kumar, 2017). For the last two decades, the scope of epigenetic studies is expanding continuously with the identification of more and more epigenetic marks (Shi et al., 2017). Identification of additional DNA base modifications (5-hmC and 6-mA), known to have epigenetic regulatory functions in animals, resulted in the increased significance of epigenomic studies. 5-mC is a well-studied epigenetic mark among the other modified nucleosides in DNA.

Epigenomic changes play an important role in the regulation of gene expression in both animals and plants during development, suppression of transposable elements (TEs) and response to environmental stresses (Chinnusamy and Zhu, 2009; Kumar and Singh, 2016; Kumar et al., 2017a). While the genome is largely consistent within an individual throughout its life, the epigenome is dynamically influenced by environmental factors and developmental processes; hence, the epigenome may vary in different cells under different environmental conditions (Shi et al., 2017). The epigenetic changes may cause variation in the structure of chromatin and expression of the genome. The epigenetic changes may result in chromatin remodeling, which may cause an alteration in transcription level even after withdrawal of the stress (Avramova, 2015). The epigenetic marks are maintained by DNA methylases (writers) and demethylases (erasers), and the reader proteins (see the section “Glossary”) read and interpret the encoded information (Torres and Fujimori, 2015; Feinberg et al., 2016). Thus, a complex interactions among the different molecular factors including DNA methylation/demethylation, enzymes responsible for post-translational modifications of histone proteins, non-coding RNAs and chromatin remodelers are accountable for the epigenetic regulation of gene expression (Lauria and Rossi, 2011; Pikaard and Mittelsten-Scheid, 2014; Gallusci et al., 2016).

DNA (de)methylation regulates activation/silencing of TEs, genomic imprinting, developmental processes and stress responses in both plants and animals (Chinnusamy and Zhu, 2009; Allis and Jenuwein, 2016; Springer and Schmitz, 2017). This review focuses on the recent advances in understanding the functional consequences of the well-studied cytosine modifications and the recent studies on epigenetic modification of adenine which appears to serve as an additional layer of epigenetic regulation.

## Epigenetic DNA Modifications

Chemical modifications of nitrogenous bases of DNA play a vital role in the regulation of gene expression. Methylated cytosine (5-mC), also known as the fifth base of DNA, was recognized long before the DNA was identified as the genetic material. While more attention is given on the conventional modified DNA base 5-methylcytosine (5-mC), the recent discovery of additional base-modifications has resulted in an increased interest in epigenomics studies. DNA base modifications have been detected in all the kingdoms of living organisms, including eukaryotes, prokaryotes, and viruses. More importantly, the dynamics of epigenetic gene regulation requires removal of the epigenetic mark. The newly discovered diversity in epigenetic modifications and the potential for their combinatorial interaction indicate that the epigenetic codons are considerably more complicated than it is considered presently (Breiling and Lyko, 2015).

## The Fifth Base of Nucleic Acid: 5-Methylcytosine

DNA contains four nitrogenous bases namely cytosine (C), guanine (G), adenine (A), and thymine (T). T is replaced by uracil (U) in the case of RNA. DNA may also contain modified bases like 5-mC, 5-hmC, 5-fC, 5-carboxycytosine (5-caC), and 6-mA in a small amount (Kumar, 2018b), while RNA contains more than 140 different types of modified bases (Sloan et al., 2017). Methylcytosine (5-mC) is most common among these modified bases in the genome, and hence it is considered as the fifth base of DNA. More than 4% of the cytosines present in the human genome have been reported to be methylated (Breiling and Lyko, 2015). However, the level of 5-mC content may vary significantly among animals and plants. The significance of the 5-mC cannot be demarcated merely by its abundance, it also depends on its positioning [in symmetric (CG and CHG) and asymmetric (CHH) contexts] as well as on its location in different parts of a gene. In the animal, 5-mC occurs predominantly in CG context, but in plants, it occurs in all the three contexts (CG, CHG, and CHH) (Wang et al., 2016). Symmetric methylation indicates that 5-mC is present in CG or CHG contexts in the antiparallel strands of DNA, and the methylation pattern can be truthfully reproduced during DNA replication. In the human genome, >80% of the cytosine present in CG context is methylated, which indicates ubiquitous methylation landscape of the genome; however, site-specific gaps are often present in the regulatory elements (e.g., enhancers and promoters) of the actively transcribed genes. On the contrary, an extremely low level of methylation has been reported in certain invertebrates like Drosophila and Silkworm (Xiang et al., 2010).

Cytosine methylation affects the accessibility of the genomic regions to regulatory proteins/protein complexes, which influences chromatin structure and/or affects the rate of transcription of the gene. Cytosine methylation is the only well-studied DNA modification with established maintenance mechanisms. It has been associated with repression of the gene when present in the promoter and enhancer regions (Charlet et al., 2016), but gene body methylation (gbM), i.e., 5-mC in the coding/transcribed region excluding the transcription start site (TSS) and transcription termination site (TTS), might repress or enhance transcriptional activity (Buck-Koehntop and Defossez, 2013; Spruijt and Vermeulen, 2014).

**FIGURE 1:**
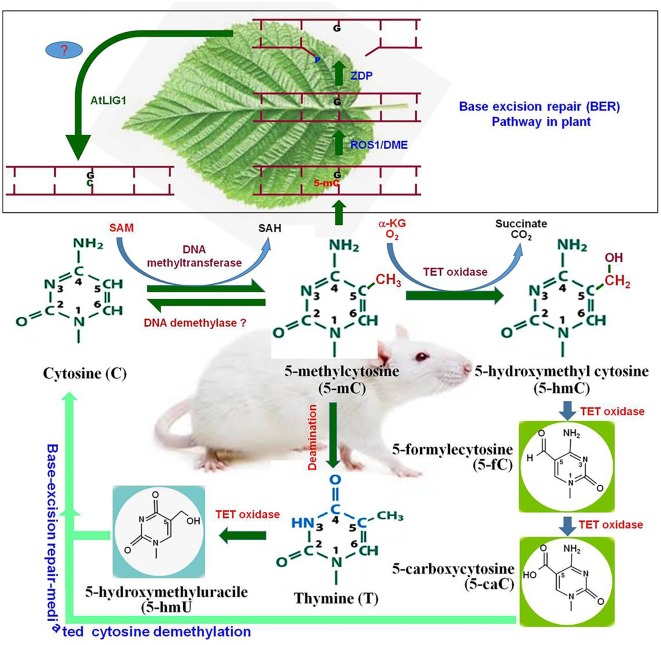
Cytosine modifications and active demethylation pathway in animal system. Cytosine is converted to 5-methylcytosine (5-mC) by DNA methyltransferase. By the action of DNA demethylase, 5-mC may get converted back to cytosine (C). Tet oxidase (Tet-1, Tet-2, or Tet-3) oxidize 5-mC to 5-hydroxymethylcytosine (5-hmC). 5-hmC can be further oxidized by Tet oxidase to 5-formylcytosine (5-fC) and subsequently to 5-carboxycytosine (5-caC). Finally, 5-caC and the deamination product of 5-mC [firstly thymine and then 5-hydroxymethyluracil (5-hmU)] are replaced by the cytosine via base-excision repair pathway. The upper pannel (in box), represents base-excision repair (BER) pathway for active DNA demethylation in plants. Repressor of Silencing (ROS1) and Demeter (DME) remove 5-mC and cleave the DNA backbone to generate a gap with 3′-phosphate terminus which gets converted into 3′-OH by Zinc finger DNA 3′-phosphoesterase (ZDP). The gap is finally filled with a usual cytosine (C) by an unknown DNA polymerase (?) and AtLIG1.

**FIGURE 2:**
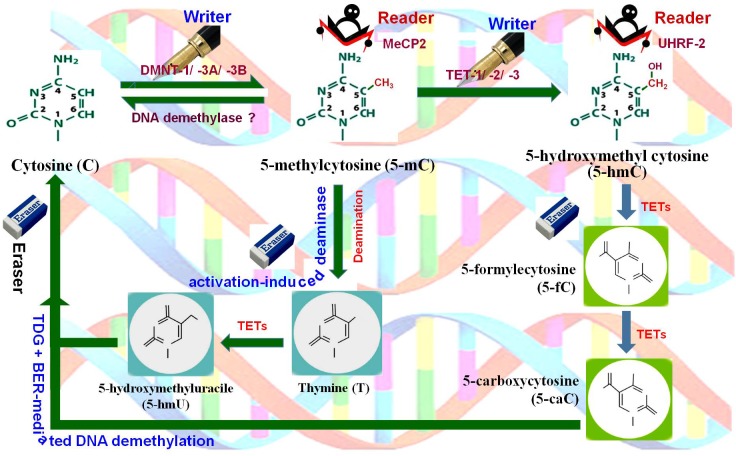
DNA (de)methylation dynamics. Cytosine (C) is methylated at 5′ carbon of the pyrimidine ring by DNA methyltransferases (DNMT-1, DNMT-3A, DNMT-3B), the writer, to generate 5-methylcytosine (5-mC), which is recognized by methyl-CpG binding protein 2 (MeCP2), the reader. 5-mC gets hydroxylated by Ten-Eleven Translocation 1 (TET-1, TET-2, TET-3) methylcytosine dioxygenases, the writer, to 5-hydroxymethylcytosine (5-hmC) which is recognized by ubiquitin-like PHD and Ring finger domain-containing proteins (UHRF-1, UHRF-2), the readers. 5-mC may also get deaminated by cytidine deaminase (activation-induced deaminase), the eraser, to generate 5-hydroxymethyluracil (5-hmU). Further, 5-hmC gets oxidized by TETs to 5-formylcytosine (5-fC) and then to 5-carboxylcytosine (5-caC). Finally, all of these intermediates are substrates for thymine-DNA glycosylase (TDG) and base-excision repair (BER)-mediated DNA demethylation, the eraser pathway.

In plants, cytosine methylation at symmetric (CG and CHG) context is maintained by methyltransferase 1 (MET1) and chromomethylase 3 (CMT3), while methylation at asymmetric (CHH) context is maintained by RNA-dependent DNA methylation pathway (RdDM) pathway or by the chromatin remodeler DDM1-dependent chromomethylase 2 (Zemach et al., 2013). Whole-genome bisulfite sequencing analysis of *Arabidopsis thaliana* revealed that gbM is limited to CG context, and methylation at CHG and CHH context is found in TE- and repeats-enriched heterochromatin regions. Methylation level varies significantly in CG and non-CG contexts. In *A. thaliana*, methylation at CG context was reported to be 24%, while methylation at CHG and CHH context was found to be 7% and 2%, respectively (Cokus et al., 2008). It was found to be 86%, 74%, and 5% at CG, CHG and CHH contexts, respectively, in maize at the reproductive stage (Gent et al., 2013). Methylation at the non-CG context in plants plays a key role in silencing exogenous DNA via the RdDM pathway (Law and Jacobsen, 2010). Therefore, it is reasonable to assume that the methylation of gene/genome is the default state, and specific mechanisms are needed to maintain specific regions free from methylation. Methylation level is dynamically controlled by DNA (de)methylation processes. DNA demethylation takes place by active and/or passive methods. During active DNA demethylation, 5-mC is oxidized to 5-hmC, 5-fC, and further to 5-caC followed by base-excision repair (BER) mechanism which is independent of replication process (Figure 1). Thus, active DNA demethylation engages enzymatic removal of 5-mC. In plants, a family of DNA glycosylases (erasers) namely DME (Demeter) ROS1 (Repressor of Silencing 1), DML2 (Demeter-like 2), and DML3 initiate active DNA demethylation, and it is completed by the BER-dependent mechanism (Zhu, 2009) (Figure 2). DME directly removes 5-mC from the DNA backbone, creates an abasic site which is filled up by the BER pathway (Li et al., 2018). Mutations in the gene(s) of DNA demethylation pathway affect DNA methylation, which leads to genome-wide DNA hypermethylation, and cause silencing/activation of the TEs/genes expression of which is no longer needed and must be repressed (Lang et al., 2017; Frost et al., 2018). Transcriptional repression of MET1 was reported to be associated with genome-wide passive DNA demethylation (Ji et al., 2014). However, it is still not known how global demethylation occurs during gametogenesis and embryogenesis in plants. BER pathway cannot be the primary process for global demethylation because this would result in so many abasic sites/broken DNA strands, which will destabilize the genome (Zhu, 2009). Thus, we can speculate that another pathway might be involved in active DNA demethylation in plants, and this unknown active demethylation pathway might be similar to the known Ten-Eleven Translocation (TET) pathway in mammals; however, such pathway has not yet been identified. Activation of genes during the developmental process, environmental stresses, and genome-wide epigenetic reprogramming are mediated by active DNA demethylation (Hsieh et al., 2009; Kumar et al., 2017b).

## Oxidation of 5-mC Creates Another Epigenetic Mark: 5-Hydroxymethylcytosine

Shortly after the discovery of 5-mC in 1948, 5-hmC was identified in a bacteriophage and vertebrates, including a frog, mouse, and rat. Hydroxylation of 5-mC in viral DNA was reported earlier to be a mechanism to tag it to differentiate from the cytosine of host DNA and to avoid BER pathway of the host defense mechanism. Though the biological significance of 5-hmC has been investigated lately (Kriaucionis and Heintz, 2009), its function as an epigenetic mark is poorly understood (Shi et al., 2017). 5-hmC is an intermediate of active DNA demethylation process, and it is a stable epigenetic mark in animals. With the discovery of enzymatic functions of TET family proteins, 5-hmC and the TET-dependent oxidation products [5-fC, 5-caC, 5-hydroxymethyluracil (5-hmU)] are considered to be the demethylation intermediates of 5-mC in animals. Interestingly, these modified-cytosine bases might act as important epigenetic marks. These epigenetic marks (5-fC, 5-caC, and 5-hmU) have been reported to play an important role in the regulation of transcription process, chromatin remodeling, and recruitment of DNA repair-associated complexes in animal (Fong et al., 2013; Yue et al., 2016). Novel epigenetic modifications of DNA are now being identified with the discovery of catalytic dioxygenase activity of the TET enzymes (Tahiliani et al., 2009; Ito et al., 2010). 5-hmC content in mammalian tissues is about 0.1% but can vary greatly (Globisch et al., 2010) with the highest level in the brain where it can go up to 1% (Kriaucionis and Heintz, 2009). Three mammalian TET homologs (TET-1, TET-2, and TET-3) are involved in the conversion of 5-mC to 5-hmC, 5-fC, and to 5-caC. While *Tet1* was found to express in the embryonic stem cells (ESCs), *Tet2* and *Tet3* exhibited a similar expression pattern in various tissues. Thus, *Tet* genes show cell/organ-specific expression (Ito et al., 2011; Tollervey and Lunyak, 2012). In mouse ESCs (mESCs), about 30000 5-mC, 1300 5-hmC, 20 5-fC, and only 3 5-caC per million cytosine residues were observed (He et al., 2011; Ito et al., 2011), which indicate the sporadic presence of 5-fC and 5-caC. These unusual modified bases (5-fC and 5-caC, and deamination products of 5-mC: thymine or 5-hmU), are removed by BER mechanism (He et al., 2011; Ito et al., 2011). Thymine-DNA glycosylase (TDG) removes T from G:T mismatches, and initiates BER of the deaminated 5-mC. TDG was found to be active on 5-fC and 5-caC, but not active on 5-mC or 5-hmC (He et al., 2011; Maiti and Drohat, 2011). Thus, cytosine methylation dynamics is managed by the activities of the DNA methyltransferases, TET enzymes and TDG pathway.

The role of 5-hmC as an epigenetic mark and the specific functions of TET enzymes in animal system are beginning to emerge. *TET1* and *TET2* genes are highly expressed in mESCs, but a depleted expression of either of these genes did not affect pluripotency or development (Dawlaty et al., 2011; Ko et al., 2011; Koh et al., 2011; Li et al., 2011). Homozygous *TET3* mutant mice showed proper embryonic development, but they died at birth (Gu et al., 2011), which suggests that TET3 is not necessary for embryonic development. Mice ESCs with defective TET1 and TET2 showed no significant change in 5-hmC level and retained pluripotency, but developmental defects associated with the ectopic hypermethylation were observed (Dawlaty et al., 2013). However, triple-mutant (*TET-1*, *TET-2*, and *TET-3*) mESCs were found to be viable, pluripotent and showed depleted 5-hmC content (Dawlaty et al., 2014; Lu et al., 2014). They showed hypermethylation of promoters and impaired differentiation potential (Dawlaty et al., 2014), which indicate a major role of active DNA demethylation via TET-mediated oxidation in maintaining the regulatory regions, particularly enhancers and promoters, free from 5-mC (Lu et al., 2014). The potential role of 5-hmC as active epigenetic mark was further supported by mass spectrometric analysis of radiolabeled DNA from mammalian and mice cells having stable 5-hmC without any transient intermediate (Bachman et al., 2014). The 5-hmC level was found to increase during neuron differentiation, and stable intragenic 5-hmC content was observed in many active neuron-specific genes (Hahn et al., 2013). The abundance of 5-hmC was found in constitutive exons compared to that in the alternatively spliced exons (Khare et al., 2012). Therefore, it is plausible to assume that 5-hmC plays a role in alternate splicing, wherein it contributes to the binding of regulatory proteins to DNA. Thus, 5-hmC may be considered as a stable modified-base found in mammalian promoters, enhancers, near the TSS, coding region of the actively transcribed genes, 3′ UTR and intragenic regions (Wu and Zhang, 2011; Pastor et al., 2013). It is also believed that 5-hmC acts as *cis*-element to repress or promote expression of the gene. Besides, 5-hmC can be associated with histone-modification which may collectively alter the configuration of chromatin as well as with switching “on” or “off” the genes in heterochromatic and/or euchromatic regions (Shi et al., 2017).

While there is evidence for epigenetic functions of 5-hmC, at least in some tissues of animals, the similar function of its oxidation-derivatives (5-fC and 5-caC) appears to be less likely. Although the presence of 5-fC at the 5-hmC - marked regions suggest its potential role as an independent epigenetic mark, yet it needs to be confirmed. The level of 5-fC and 5-caC was found to increase in thymine-DNA-glycosylase-deficient mESCs, which suggests that 5-caC represents the site of active DNA demethylation (Raiber et al., 2012; Song et al., 2013). It has also been observed that TET proteins can oxidize thymine (particularly those produced by deamination of 5-mC) to 5-hmU which enters into BER pathway (Figure 1) (Pfaffeneder et al., 2014). Though some of the reports suggest the existence of 5-hmC in plants, they have conflicting inferences. Erdmann et al. (2015) investigated the existence of 5-hmC in *A. thaliana* and other plants using various techniques like thin-layer chromatography, ELISA, ChIP-chip, enzymatic-radio-labeling, and mass spectrometry. While the antibody-based techniques suggested a lower level of 5-hmC in the plant genome, the most sensitive techniques like enzymatic-radio-labeling and mass spectrometry failed to detect it in genomic DNA from different tissues. The findings suggest that 5-hmC may not be present in biologically relevant quantity in the plant genome (Erdmann et al., 2015). Studies on three rice cultivars indicated that 5-hmC is present at very low quantity (1.39 to 2.17 per million nucleosides) especially in TEs and heterochromatin regions (Wang et al., 2015). Moreover, the occurrence of 5-hmC was detected in the inactive TEs.

Neither TET proteins nor UHRF2 [Ubiquitin-like, PHD and Ring Finger Domains 2, which specifically recognize 5-hmC, the reader (Figure 2)], well characterized in the animal system (Zhou et al., 2014), have been identified yet in plants. Therefore, it is speculated that DNA demethylation is not processed through TET pathway in plants. It is not yet clear that 5-hmC is implicated in what type of cellular processes in the animal system, and its epigenetic relevance is yet to be understood (Klungland and Robertson, 2017). Hence, existence and epigenetic role of 5-hmC, if any, is yet to be discovered in plants (Terragni et al., 2012; Moricová et al., 2013; Wang et al., 2015). These findings rejuvenate interest in understanding the epigenetics of 5-hmC, and hence 5-hmC is now considered as the sixth base of the epigenome (Shi et al., 2017).

## Non-Cytosine Methylation of DNA: N^6^-Methyladenine

**FIGURE 3:**
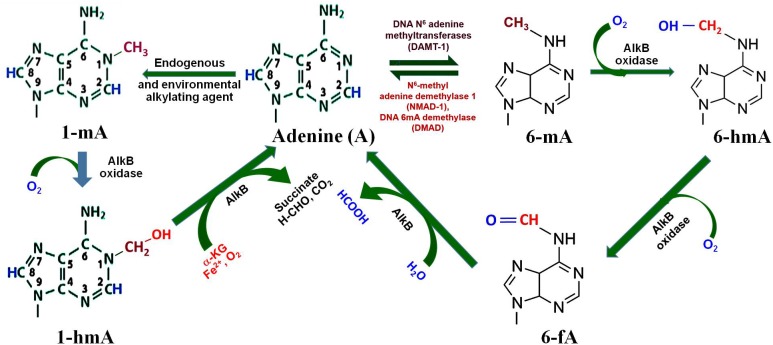
Adenine modification dynamics. Adenine gets methylated by DNA N^6^-adenine methyltransferases (DAMT-1) to produce N^6^-methyladenine (6-mA), as observed in *C. elegans*. 6-mA can be demethylated by the action of N^6^-methyl adenine demethylase-1 (NMAD-1) or DNA methyladenine demethylase (DMAD). Oxidation of methyl group of 6-mA by AlkB oxidase results in the formation of N^6^-hydroxymethyl adenine (6-hmA) and N^6^-formyl adenine (6-fA), and finally back to adenine. Adenine may also get methyl adduct by endogenous or environmental alkylating agents to N^1^-methyladenine (1-mA), which may further get demethylated by AlkB oxidase to adenine via N^1^-methylhydroxy adenine (1-hmA).

Methylation of adenine, i.e., 6-mA, in GATC context has been reported for the survival of several bacteria, as DNA adenine methyl transferase (DAMT, the writer) creates specific methylation marks (Figure 3) that are important for DNA replication, mismatch repair, segregation, and regulation of gene expression (Ratel et al., 2006). 6-mA is known to play an important regulatory role in RNAs, and recent studies suggest the presence of 6-mA in eukaryotic genomes (Kigar et al., 2017; Yao et al., 2017, 2018). Moreover, oxidation of the methyl group of 6-mA by AlkB family of dioxygenases (e.g., 6-mA demethylases) results in the formation of 6-hmA and 6-fA, which can restore the original base (Figure 3) by releasing formaldehyde (Fu et al., 2013). A recent study on *Caenorhabditis elegans* demonstrated that NMAD-1 (an AlkB family enzyme) demethylates 6-mA in DNA (Fu et al., 2015). The study suggests that the enzymes of the AlkB family are critical players in demethylation of 6-mA in DNA (Iyer et al., 2016). Adenine may also get methylated to N^1^-methyladenine (1-mA) by the endogenous or environmental alkylating agents (Sedgwick et al., 2007), which may get demethylated indirectly by AlkB oxidase to adenine via N^1^-methylhydroxy adenine (1-hmA).

In *Escherichia coli*, 1-mA is generated due to the environmental alkylation agents which creates a local lesion, prohibits the formation of regular Watson–Crick base pairing, and consequently blocks DNA replication (Sedgwick et al., 2007). *AlkB* is an inducible gene in *E. coli* for adaptive response to the *alk*ylating agents. Human AlkB homologs perform similar base modifications and exhibit overwhelming functional roles (Westbye et al., 2008). Similarly, N^7^-methylguanine (7-mG) is also produced due to alkylation by the endogenous and/or environmental alkylating agents. A comprehensive understanding of these base modifications and their functional roles would be necessary to discover their contribution, if any, in epigenetic regulation of gene expression in animals and plants.

**FIGURE 4:**
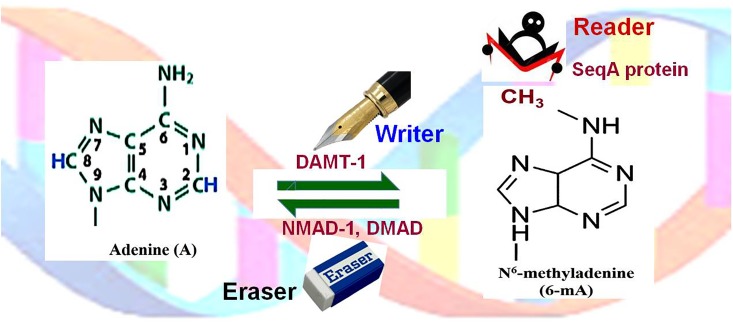
Adenine (de)methylation dynamics. Adenine (A) is methylated at N6 of the purine ring by DNA N^6^-adenine methyltransferases 1 (DAMT-1), the writer, to generate N^6^-methyladenine (6-mA). SeqA protein, the reader, preferentially binds to hemimethylated (6-mA) DNA. 6-mA may get demethylated by the action of N^6^-methyl adenine demethylase-1 (NMAD-1) or DNA methyladenine demethylase (DMAD), the eraser.

In several unicellular eukaryotes, including *Chlamydomonas reinhardtii*, comparatively higher level of 6-mA has been reported (Ratel et al., 2006). Several powerful techniques have been developed for detection of 6-mA, and this has resulted in the identification of some key features of 6-mA in *Chlamydomonas* (Fu et al., 2015). The algal adenine-methylome consists of about 85000 6-mA in AT sequence context, mainly in the promoter and the linker regions. Therefore, it was proposed that 6-mA marks the position of nucleosomes near TSS (Fu et al., 2015). Moreover, *Chlamydomonas* genome has been characterized to have a low level of cytosine methylation in CG, CHG, and CHH contexts in the genes, which corroborates with the methylation pattern in plants (Wang et al., 2016). *C. elegans* and *Drosophila melanogaster* possess the negligible amount of 5-mC or 5-hmC, but the significantly high level of 6-mA. In these organisms, such DNA base modification is considered as a new epigenetic mark. Experimental data from *C. elegans* suggest a functional correlation between 6-mA and a histone modification H3K4me2 (Greer et al., 2015). In *D. melanogaster*, mutations in DNA 6-mA - demethylase (DMAD, a TET-homolog) caused increased TE activity (Zhang et al., 2015). In these organisms, mutations in DMAD (eraser) caused significant phenotypic aberrations including developmental defects and infertility, suggesting epigenetic functions of 6-mA. SeqA protein serves as a reader of 6-mA (Figure 4), preferentially binds to hemimethylated DNA, and affects gene expression/chromatin activity. Therefore, future research needs to discover the functions of the newly identified epigenetic marks (5-hmC, 5-fC, 5-caC, 5-hmU, and 6-mA), enzymes that are involved in setting up (writing) and removing (erasing) these modifications, and the reader that recognizes/binds the epigenetic mark.

Report on the occurrence of 6-mA in higher eukaryotes is sparse, and the finding has been often inconclusive (Ratel et al., 2006). Yao et al. (2017) demonstrated the dynamics of 6-mA in mouse brain under environmental stress. They reported that the levels of 6-mA get significantly elevated upon the stress. They also observed an inverse relationship between 6-mA dynamics and expression of certain upregulated neuronal genes or downregulated transposons. However, the presence and function of 6-mA in the mammal remain unclear. Highly sensitive mass spectrometry technique detected less than one 6-mA per million nucleotides in mouse genomic DNA (Fong et al., 2013). This suggests that 6-mA is either not a common (like 5-mC/5-hmC) modified base or its turnover by demethylation process is very rapid. However, accumulation of 6-mA by deactivating DMAD has been demonstrated in *D. melanogaster* (Zhang et al., 2015). It has also been found that demethylation of 6-mA is mediated by a TET-like enzyme in *D. melanogaster* (Zhang et al., 2015). Their finding indicates that cytosine and adenine (de)methylation occur in a coordinated, dynamic and sequence context-specific manner. Recently, Yao et al. (2018) reported epigenetic regulation of a group of genes involved in neurodevelopment and neuronal functions in *D. melanogaster*. Accumulation of 6-mA due to deactivated DMAD coordinates with Polycomb proteins and contributes to transcriptional repression of the genes. Hence, it would be interesting to investigate the interplay between different epigenetic marks to explore the complexity of the epigenetic code, which might answer several biological enigmas in the near future (Kumar, 2017).

Study on *C. elegans* revealed that about 0.3% adenine are methylated in GAGG and AGAA sequence contexts. Interestingly, 6-mA was observed to accumulate in *C. elegans* deficient for *spr-5* (codes for H3K4me2 demethylase) (Greer et al., 2015). While 5-mC increases DNA helix stability, 6-mA destabilizes the helical structure of DNA. Therefore, 5-mC is considered to be a repressor of transcription process when it occurs in the promoter region, and 6-mA functions as an activator of transcription.

## Advances in Detection of Modified DNA Bases

Epigenetic changes are reported to be important players in determining cellular growth, development, differentiation processes, and tolerance to abiotic and biotic stresses in the organisms. There is evidence suggesting an association between variations in DNA methylation and environmental perturbations in animals and plants. Therefore, detection of epigenetic changes has become critically important for scientific/diagnostic purposes. Information about alterations in epigenetic marks might be useful in identifying the stress level and the associated problems, such as the occurrence of cancer in an animal might be detected right at the early stage of development. One of the exciting technological advancements in the field of epigenomics has been the invention of different efficient methods having certain advantages and disadvantages to detect/analyze DNA base modifications (Cokus et al., 2008; McIntyre et al., 2017). Considerable progress is being made in the identification/quantification of the modified DNA bases, which has significantly broadened the area of epigenomics research.

### Detection of 5-mC

To detect methylcytosine (5-mC) at the genome level, methylation-sensitive amplified polymorphism (MSAP) technique has been being used, which uses a modified protocol of amplified fragment length polymorphism (AFLP). In MSAP, isoschizomers *Hpa*II and *Msp*I are used at the place of *Mse*I (a frequent cutter), while the hexa-cutter *Eco*RI remains the same (Xiong et al., 1999). Though *Hpa*II and *Msp*I have the same restriction site (5′-CCGG-3′), they show differential sensitivity to 5-mC. This is why the genomic profile generated using MSAP presents the variations in methylation at the enzyme’s recognition site only. Another technique being used for quantification of 5-mC at whole-genome level utilizes monoclonal antibody specific to 5-mC. This requires a smaller amount of genomic DNA, provides reliable results at the lower cost and higher throughput compared to those obtained by HPLC based detection of 5-mC. Several efficient methods have been devised to determine 5-mC content at single-base resolution, and most of them take advantage of sodium-bisulfite modification reaction which deaminates cytosine (but not the 5-mC) to uracil (U). This results in the conversion of C to thymine (T) in DNA during the synthesis of the complementary strand.

This allows to distinguishing unmethylated cytosine (C) from 5-mC. Combination of bisulfite conversion and high-throughput DNA sequencing for determining DNA methylation status is referred to as whole-genome bisulfite sequencing (WGBS). Presence of C in the sequencing read typically indicates that the cytosine was 5-mC, as the 5-mC is protected from conversion by the sodium bisulfite treatment. This allows determination of the content, location, and context (CG, CHG, CHH) of 5-mC for better interpretation of the results. This also generates quantitative data for DNA (de)methylation at the individual site because of the multiple independent reads that are aligned with the reference sequence. Although WGBS cannot distinguish 5-mC from 5-hmC, yet there are chemistries to resolve this problem. Moreover, certain variations in bisulfate sequencing often affect the detection/measurement of 5-mC in tissue samples, which may differ even within the same epigenome-wide association studies (EWAS). Variants like bisulfite-conversion efficiency, DNA polymerase efficiency/fidelity, number of cycles of PCR, amount of the template DNA used, bias due to Gibbs law (more CG sites on probe = greater hybridization, regardless of modification), the DNA strand (leading, lagging or both) used to read, the number of reads performed during sequence, etc. affect detection efficiency of the technique. For example, in case of Illumina 450K only 500 ng DNA is required, >100 cycles of amplification are performed using an enzyme with much lower fidelity (compared to that of the high-fidelity enzyme like Phusion DNA Polymerase having 50× higher fidelity than Taq DNA polymerase and 6× lower Pfu DNA Polymerase). All of these cause variations lead to misinterpretation of the results, unable us to compare data from different studies, and reduce reproducibility of data even when the same tissue and technology is used. Therefore, in addition to focusing on epigenomics of single cell type and increasing data sets, methylation data should always be confirmed by more than one assays (e.g., bisulfite-pyrosequencing, MeDIP-qPCR, MS-qPCR, RRBS (reduced representation bisulfate sequencing), etc.), which would provide a more balanced, comprehensive and critical view of the researchable issue.

Recently, nanopore sequencing has improved the read-length (10-100 kb), sequencing throughput, and more importantly enabled direct detection of DNA base modifications. Nanopore sequencing method has been utilized to detect DNA base modifications (such as 5-mC) based on the sensitivity of electrolytic current signals to base modifications. Simpson et al. (2017) reported quantification of 5-mC using this effect in the Oxford Nanopore Technologies MinION sequencer to sequence the human methylome. Further advances in the nanopore sequencing are expected to revolutionize epigenomic studies through high throughput next-generation sequencing.

### Detection of 5-hmC

Classical methods for quantification of 5-hydroxymethylcytosine (5-hmC) have been based on sophisticated instruments such as liquid chromatography, mass spectrometric techniques (LC-MS, HPLC-MS). More recently, quantification of 5-hmC based on immunoassay principles (utilizing antibodies specific to 5-hmC) has been commercialized. Although, these methods are quantitative and reproducible; they are complicated, expensive, lack sensitivity, and they are less suitable for high throughput analysis. Thus, several methods for detection and quantification of 5-hmC do exist, yet none of these techniques meet the requirements of advanced epigenetic studies. Besides, a larger amount of DNA is required when the sample contains a lower level of 5-hmC. Moreover, the detection limit of these techniques is approximately 0.03% 5-hmC per dNTP which further limits their utility. Interestingly, these techniques provide only a relative value for 5-hmC content; hence, for absolute measurement of 5-hmC a calibration curve needs to be generated first.

Another method utilizes phage T_4_ β-glucosyltransferase (β-GT) enzyme which catalyzes attachment of β-D-glucosyl residues of uridine diphosphoglucose (UDP-Glu) with the hydroxyl group of 5-hmC. In this method, a reactive azide group is used to label 5-hmC, and the azide group is subsequently tagged with a fluorescent-labeled strained alkyne like dibenzocyclooctyne-Cy5 reporter molecule. Although this is an accurate and high-throughput quantification method for quantification of 5-hmC, a larger amount of DNA (∼6 μg) is required for the estimation. Hence, to minimize the amount of DNA sample required for detection of 5-hmC, the ultra-sensitive single-molecule approach was utilized by Gilat et al. (2017).

### Detection of 5-fC and 5-caC

Genome-wide analysis of 5-fC in mESCs revealed preferential occurrence of 5-fC at enhancers among the other gene regulatory elements. 5-fC exhibits a preference to the poised enhancers, suggesting a role for 5-fC in epigenetic priming of enhancers (Song et al., 2013). Accumulation of 5-caC at promoter regions of several genes in hepatic cells at differentiation corresponds with the beginning of their expression. Since 5-fC and 5-caC show similar behavior to cytosine in bisulfate sequencing and occur at very low abundance in mammalian genomic DNA (Ito et al., 2011), their detection becomes a challenging task using antibody-based immunoprecipitation technique. Song et al. (2013) used 5-fC-selective chemical labeling approach and a 5-fC chemically assisted bisulfite sequencing method for genome-wide profiling of 5-fC. Wheldon et al. (2014) and Lewis et al. (2017) reported the use of 5-caC-DNA immunoprecipitation followed by bisulfite sequencing for genome-wide profiling of 5-caC in mESCs.

### Detection of 6-mA

Initially, the presence of N^6^-methyladenine (6-mA) in bacteria was confirmed by using a combination of the ultraviolet absorption spectrum, electrophoretic mobility, and paper chromatography. These methods are relatively insensitive, hence the presence of 6-mA in eukaryotes is not detectable. Later on, researchers realized that restriction enzymes can be utilized to identify 6-mA residues. However, limitations of this approach are the dependence of the restriction enzyme on occurrence of 6-mA in the appropriate restriction enzyme target motif. Therefore, this method cannot detect the presence of 6-mA in different sequence contexts. High-performance liquid chromatography (HPLC) was then used to detect 6-mA in *E. coli*.

Subsequently, liquid chromatography and recently ultra-high performance liquid chromatography combined with mass spectrometry (UHPLC-MS/MS) are used to detect 6-mA. While the above-mentioned techniques have been proven useful for detection of the presence of 6-mA, they do not provide any information about its genomic location. When methylated DNA immunoprecipitation (MeDIP) technique is coupled with UHPLC-MS/MS, which can distinguish 1-mA from 6-mA, it can give unambiguous confirmation about the genomic location of 6-mA (Greer et al., 2015). Therefore, to convincingly identify 6-mA a combination of complementary techniques would be essential, as each technique has its own set of limitations. Recently, McIntyre et al. (2017) used nanopore sequencing to detect 6-mA in bacteria deploying a new generation base-caller method (nucleic acid modification caller, mCaller) to detect the base modification.

However, the pattern of DNA modifications are dynamic in nature during cellular differentiation and development stages with introduction and/or erasing of different sets of base modification(s) in the genomic regions specific for the developmental stage. Cellular differentiation appears to be governed by methylation homeostasis in the mammalian genome. However in epigenetic research, the challenge has been in linking specific epigenetic and environmental factors to cellular function/behavior. In fact, most of the research findings discussed in this review is merely based on correlation analysis using mixed cell populations often without any attempt to functionally confirm the linkage between the epigenetic mark and the change in expression of a gene in the cell/tissue. Merely because an epigenetic variation has been found to appear with a change in phenotype, it does not necessarily mean that the phenotypic change is because of the epigenetic action. Generally, the normal cell growth, development and pathology in plants and animals are based on the multi-dimensional information. Therefore, the need of the day is to integrate epigenetic information with phenotypic change using modern techniques like optogenetics (Guru et al., 2015), sensor-based live imaging (Ingouff et al., 2017) epigenome editing (Liao et al., 2017), etc. While optogenetics uses light to control cells in living tissue to monitor the expression of light-sensitive ion channels in genetically modified cells, the sensor-based live imaging provides insight into global DNA modification dynamics at single-cell level with a high temporal resolution during development stages and in response to environmental stress. On the other hand, epigenome editing aims at targeted *in vivo* editing of the genomic region(s) without altering DNA sequence using the tools like CRISPR-dCas9.

Recently, 6-mA was identified in *Oryza sativa* and *Zea mays* utilizing more sensitive detection methods like HPLC-MS/MS (Huang et al., 2015). DRM2 has been shown to catalyze both cytosine- and adenine-methylation in Arabidopsis, but in wheat N^6^-adenine DNA-methyltransferase was reported to perform this job. The data on 6-mA in plants must be viewed with caution unless it is validated that the detected 6-mA was not from mitochondrial DNA of the plant or from contaminating symbionts. It has been observed that the organisms having a higher content of 6-mA (e.g., bacteria and unicellular eukaryotes) generally have a lower content of 5-mC, while the organisms with higher content of 5-mC (e.g., plants and mammals) possess a lower content of 6-mA (O’Brown and Greer, 2016). If 6-mA is found to be present in a significantly higher quantity in eukaryotes also, it might turn out to be the seventh base and carrier of the epigenetic information in living organisms.

## Conclusion and Future Perspectives

Epigenetic modifications influence the accessibility of the genomic regions for regulatory proteins, which affect chromatin structure, enhancer/promoter activity, and the transcriptional process. Cytosine modifications, particularly methylation, are the known epigenetic changes with established maintenance mechanisms and some of the functions which enable their use as an epigenetic mark (Kumar, 2018a). Cytosine methylation has generally been associated with repression of gene expression when present in prompter regions, but it might also enhance the gene expression either by recruiting methylation-specific transcription factors (Buck-Koehntop and Defossez, 2013) or by yet to be discovered mechanisms (Baubec et al., 2015; Kumar et al., 2017c). The dynamic epigenetic regulation also requires removal of the modified base, which involves DNA demethylases and TET proteins. Though 5-hmC has been identified as an intermediate product of the active demethylation process, and a potential epigenetic mark in metazoan animals, its existence in plant is still controversial. Until recently, 6-mA has been thought to be present in protozoans (bacteria, archaea, and protists); however, its recently discovered existence in eukaryotes, including animals and plants, indicates that 6-mA might serve as an additional epigenetic mark. In *C. elegans*, this novel epigenetic mark shows a functional interaction with the established histone mark (H3K4me2), and in *D. melanogaster* it supports increasing transposon activity.

Nevertheless, reports on the existence of 6-mA in eukaryotes have been sparse, and often inconclusive (Ratel et al., 2006). Future research needs to focus on investigating the conserved epigenetic marks, the enzymes involved in setting, recognizing and removing these modifications, and their heritable components if any. Interestingly, 6-mA demethylation was observed to be mediated by a TET-like enzyme in *D. melanogaster* (Takayama et al., 2014), which indicates interplay between cytosine and adenine methylation/demethylation. Therefore, further studies should also focus on unraveling the correlation between the known epigenetic marks, which may render clues on their biological significance and evolutionary roles (Kumar, 2018b).

Genome editing technologies, such as CRISPR–Cas9, are continuously being improved for targeted edit purposes. Deactivated endonuclease Cas9 (dCas9) has transformed synthetic biology platforms to achieve desired gene regulation, genome editing and fluorescent labeling (Xu et al., 2016). Discovery of flexibility in sgRNA has created the possibility of inserting additional RNA elements (Konermann et al., 2015) which can be recognized by RNA-specific binding protein effectors leading to improved efficacy of targeted dCas9-mediated functional moieties (Zalatan et al., 2015). By modulating DNA (de)methylation level at specific sites, we may explore how (de)methylation at CG sites impact cellular biochemistry, physiology, and pathology. Thus, RNA-guided dCas9 (de)methylation would have versatile and broader applications in basic research as well as therapeutics. This technology might soon be improved to the point that plant epigenomes could also be manipulated, and in the coming years, we may witness epigenome engineered crop plants for certain desirable traits. However, it will be essential to identify the epigenetic mark(s) linked with the trait of interest and understand the epigenetic machinery so that we can deploy epigenetic engineering to solve clinical problems as well as to tailor make the crop plants to cope up with the deleterious effects of global climate changes.

## Author Contributions

SK and TM conceived the review. SK and VC prepared the manuscript. SK, VC, and TM revised the manuscript and approved the final draft.

## Conflict of Interest Statement

The authors declare that the research was conducted in the absence of any commercial or financial relationships that could be construed as a potential conflict of interest.

## Appendix

### Glossary

#### Active DNA Demethylation

It involves removal of methylcytosine (5-mC) and replacement with cytosine (C) using enzymatic machinery.

#### Asymmetric Context

When a cytosine (C) occurs in a sequence context (e.g., CHH, where H = A, C or T) which is not the same in the complementary strand, then it is named as asymmetric context.

#### Base Excision Repair

This is the mechanism which repairs DNA damages caused due to alkylation, deamination, and oxidation. DNA glycosylase initiates the process by recognizing and removing the damaged base, leaving an abasic site, which is subsequently repaired by another enzyme complex.

#### Chromatin

It is constituted of packaged nucleosomes (DNA wrapped around a bundle of histone proteins) and plays a vital role in the regulation of gene expression.

#### CRISPR–dCas9

The bacterial defense system based clustered regularly interspaced short palindromic repeats (CRISPR) along with the deactivated CRISPR-associated protein 9 (dCas9) constitute one of the advanced (epi)genome editing tools.

#### DNA Demethylation

This is the process of removal of methyl group from a nucleotide base, which may occur either through the active or passive mechanism.

#### DNA Methyltransferase

It is a family of enzymes involved in catalyzing the transfer of methyl (CH_3_) group to DNA base using *S*-adenosyl methionine (SAM) as a methyl group donor.

#### Epigenetics

This is the study of functionally relevant modifications (affecting gene expression) in the genome without any change in the nucleotide sequence.

#### Epigenetic Code

It is hypothesized to be the combinations of epigenetic modification(s), including DNA and histone modifications, which affect the expression of the gene.

#### Epigenetic Eraser

A group of enzymes that catalyze the removal of an epigenetic mark.

#### Epigenetic Mark

A feature not directly related to the change in genetic information (nucleotide sequence), but depends on the epigenetic modifications (DNA base and histone modifications) affecting gene expression.

#### Epigenetic Reader

A protein or protein complex that recognizes and binds to the epigenetic modification to affect the expression of the gene.

#### Epigenetic Writer

The enzyme or enzyme complex that catalyzes and establishes a particular epigenetic modification.

#### Epigenome

The sum total of chemical modifications in DNA bases (without affecting the nucleotide sequence), histone proteins and biogenesis of non-coding RNAs in a cell constitutes the epigenome.

#### Euchromatin

The loosely packaged, gene enriched part of the chromatin that is actively transcribed as per the needs is called euchromatin.

#### Gene Body Methylation

It refers to methylation of DNA base(s) present in the transcribed/coding region of the gene.

#### Genomic Imprinting

Generally the genes inherited from both the parents are equally active/expressed, but due to genomic imprinting (an epigenetic phenomenon) set of genes inherited from one of the parents are exclusively expressed.

#### H3K4me2

Indicates that the fourth lysine (K) residue of histone protein H3 is dimethylated (me2).

#### Heterochromatin

Heterochromatin is a tightly packaged form of chromatin, contains repetitive DNA sequences, devoid of a functional gene, and a rarely transcribed portion of the genome.

#### Histone Proteins

Highly alkaline proteins found in the nucleus of a eukaryotic cell that helps to package DNA into structural units nucleosomes.

#### Histone Modification

It includes post-translational modifications such as acetylation, methylation, phosphorylation, sumoylation, and ubiquitylation of particular amino acid of specific histone proteins.

#### Methylome

The sum total of methylated DNA bases in the genome of a particular cell/tissue under specified conditions or the stage of development.

#### Nucleosome

It is the preliminary structure in the packaging of DNA in eukaryotes, wherein 146 base pairs of DNA is wound around a bundle of eight histone proteins.

#### Passive DNA Demethylation

It refers to the removal of 5-methylcytosine (5-mC) from DNA during the process of replication, particularly in absence of the maintenance methyltransferases.

#### Post-translational Modification

Modification of protein or its amino acid which occurs after translation of the protein.

#### Symmetric Context

When cytosine (C) occurs in a context (e.g., CG and CHG, where H = A, C or T) having the same reading sequence on the antiparallel strands of DNA, it is called symmetric context.

#### Whole-Genome Bisulfite Sequencing

It is a next-generation sequencing technique used to determine DNA methylation (5-mC) status at single-base resolution after treating the genomic DNA with sodium-bisulfate [that converts unmethylated cytosine (C) into uracil (U)] followed by whole genome sequencing.
